# Resin Composites Reinforced by Nanoscaled Fibers or Tubes for Dental Regeneration

**DOI:** 10.1155/2014/542958

**Published:** 2014-05-27

**Authors:** Xiaoming Li, Wei Liu, Lianwen Sun, Katerina E. Aifantis, Bo Yu, Yubo Fan, Qingling Feng, Fuzhai Cui, Fumio Watari

**Affiliations:** ^1^Key Laboratory for Biomechanics and Mechanobiology of Ministry of Education, School of Biological Science and Medical Engineering, Beihang University, Beijing 100191, China; ^2^College of Engineering, University of Arizona, Tucson, AZ 85721, USA; ^3^Department of Orthopedics, Zhujiang Hospital of Southern Medical University, Guangzhou 510282, China; ^4^State Key Laboratory of New Ceramics and Fine Processing, Tsinghua University, Beijing 100084, China; ^5^Department of Biomedical Materials and Engineering, Graduate School of Dental Medicine, Hokkaido University, Sapporo 060-8586, Japan

## Abstract

It has been stated clearly that nanofillers could make an enhancement on the mechanical performances of dental composites. In order to address current shortage of traditional dental composites, fillers in forms of nanofibers or nanotubes are broadly regarded as ideal candidates to greatly increase mechanical performances of dental composites with low content of fillers. In this review, the efforts using nanofibers and nanotubes to reinforce mechanical performances of dental composites, including polymeric nanofibers, metallic nanofibers or nanotubes, and inorganic nanofibers or nanotubes, as well as their researches related, are demonstrated in sequence. The first purpose of current paper was to confirm the enhancement of nanofibers or nanotubes' reinforcement on the mechanical performances of dental restorative composite. The second purpose was to make a general description about the reinforcement mechanism of nanofibers and nanotubes, especially, the impact of formation of interphase boundary interaction and nanofibers themselves on the advanced mechanical behaviors of the dental composites. By means of the formation of interface interaction and poststretching nanofibers, reinforced effect of dental composites by sorts of nanofibers/nanotubes has been successfully obtained.

## 1. Introduction

**Figure 1:**
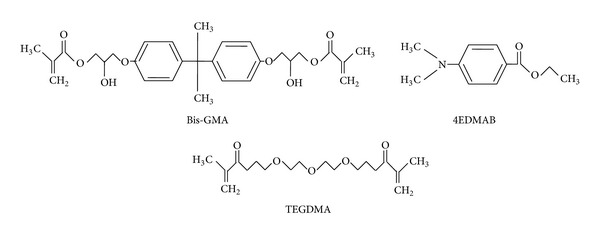
The molecular formula of common dental monomers and initiator.

In the past few decades, as a result of safety and esthetic consideration, dental composites have been generally accepted to substitute conventional “dental amalgams.” The curing mode of dental composites is free radical polymerization induced by light. Generally speaking, we usually take Camphorquinone as common photoinitiator and ethyl-4-(dimethylamino) benzoate (4EDMAB) (Figure 1) as common coinitiator. Due to the excellence of dental restorative material in safety, esthetics, operation, and workability compared with amalgam, dental resin composites can be used extensively as dental restorative material for recent decades. 2,2′-Bis-[4-(methacryloxypropoxy)-phenyl]-propane (Bis-GMA) (Figure 1) is absolutely the most commonly used polymer monomer for dental resin composites [1, 2]. In order to increase the processing performance of Bis-GMA, we usually add extra polymer monomer such as tri (ethylene glycol) dimethacrylate (TEGDMA) (Figure 1) to dilute the viscosity of resin composites. So in this multiphase resin system, Bis-GMA plays a vital role in reducing volumetric shrinkage (created in the course of the photopolymerization), boosting reactivity of the composites and increasing conversion rate of double bond to some extent [3]. Small polymerization shrinkage, good processing operation, fatigue resistance, high fracture toughness and compressive strength, low heat conduction coefficient, and the capacity to meet the requirements of the aesthetics for the teeth are some vital characteristics for desired dental composites. According to a report in 2005, the percentage of all anterior tooth direct restorations and all posterior tooth direct restorations fabricated by the dental composites accounts for more than 95% and 50%, respectively [4]. Although comprehensive performances of dental resin composites have already improved evidently, high polymerization shrinkage and lack of fracture toughness restrict the applications of dental resin composites to a great extent. What is more, it is just for the above reasons that the service life of dental resin composites is relatively shorter compared with amalgam [4–7]. To some extent, amalgam could be replaced by dental resin composites. For example, high copper amalgam and light-cured resin composites were studied on the effect of repairing the defects of posterior tooth by Li and Liu. They selected 58 subjects at age from 28 to 64 and obtained 130 posterior teeth totally. Then they classified these teeth into 2 groups: silver amalgam fillings (68 teeth) and resin material fillings (62 teeth). Experiment method was to fill the teeth in regular ways and check the filling effect after a year. Clinical results showed that light-cured resin composites outperformed in the terms of color, artistcity, and preventing microleaking [8]. Qin and Zhu made a research on 218 teeth (with different cavities) from 69 old people. They classified these teeth into two groups: P60 resin composite fillings (109 teeth) and traditional amalgam fillings (109 teeth). Two years later, they made a comparison on the effect. Results showed that repairing success rate of P60 resin composite fillings reached 94.5% and that of traditional amalgam filling reached 83.5%. In conclusion, the overall repair effect of P60 resin composite on the I and II cavities is fully satisfactory [9]. Generally speaking, the fillers in size of several microns are applied to strengthen dental resin composites. However, when dental resin composites strengthen by fillers in level of micron are under high stress circumstances, such as in case of replacement of cusps, it is these defects of dental composites that make them unsuitable for broader use. So the deficiency in intensity and service life of the composites has restricted their uses seriously [10]. The necessary condition of fillers which are added into dental composites to reinforce the material shall possess three intrinsic performances. Firstly, the fillers are substantially tougher compared with resin matrices. When the dental composites are under stress, most of the stress transfers to the resins matrices by means of kinds of specific fillers. Moreover, uniform dispersion of fillers throughout the matrices is needed. Provided that the shape of fillers immersed in resin matrices is in ordinance, the odd of appearance of stress concentration tends to be larger in the body of dental composites, and such case inclines to produce microcracks all around the fillers, thus making the dental composites weakened. Fillers highly aligned along certain orientation could impede the growth of crack, which boost up the mechanical properties of dental resin composites, thereby reinforcing the dental composites. Most importantly, in the course of preparing composite materials, what we should consider most is the interactions between fillers and resin matrix, namely, interfacial adhesion force. In the case of filler-strengthened dental composites, interfacial properties have a crucial impact on the performances of resulting dental composites. High fillers surface area could raise the extent of entanglement between various fillers and surrounding dental resins and the best case is the formation of the mutual permeating structure in the middle of fillers and the dental matrix. At the same time, powerful interfacial binding force between different phases is prospected to be produced, and then corresponding mechanical performances of resultant dental composites will be reinforced.

In the academia and industry, nanofibers and nanotubes have already been employed to strengthen dental composites. Introduction of nanofibers or nanotubes allows obtaining targeted materials with high performance in various fields. Nanofillers are very different from traditional fillers due to their large specific surface area, high aspect-ratio and unique microstructure [11]. Additionally, many researchers have proved that the application of nanotechnology could greatly improve the mechanical performances of dental composites [12, 13]. In order to offset the existing shortcomings of traditional dental composites, nanofibers or nanotubes fillers with low contents are broadly regarded as ideal candidates to greatly increase mechanical performances of dental composites. Nanofillers in the form of nanofibers/nanotubes are perfect media to disperse in the matrix uniformly [14]. Huge surface area and interfacial gliding of nanofillers in the resin matrix may give a relatively good interpretation on mechanism of damping enhancement [15–17]. Moreover, nanofillers have great potential to interact intensively with ambient polymer chains as a result of nanofillers' surface activity [18]. Also, it is known that nanofibers/nanotubes possess specific interfacial properties, which is of great importance to improve the performances of dental composite materials [12, 19]. In spite of reinforcement of natural nanofillers [20–23], we mainly consider common and feasible nanofibers/nanotubes in this paper.

Based on the above information, for the sake of satisfying increasingly high requirement for dental resin composites' mechanical performances, the research was conducted to make a further confirmation about the enhanced effects of nanofillers in forms of nanofibers/nanotubes. In this review, the methods using nanofibers and nanotubes to reinforce mechanical performances of dental composites are demonstrated in sequence, including inorganic nanofibers/nanotubes, polymeric nanofibers/nanotubes, and metallic nanofibers. The first purpose of current paper is to confirm the enhancement of nanofibers or nanotubes' reinforcement on the mechanical performances of dental restorative composite. The second purpose is to make a general description about the reinforcement mechanism of nanofibers and nanotubes, especially, the influence of formation of the mutual permeating structure and nanofibers themselves on the mechanical performances of the dental composites.

## 2. Reinforcement of Nanofibers

### 2.1. Inorganic Nanofibers

#### 2.1.1. Glass Nanofibers

Generally, inorganic fillers with a certain mass proportion are usually employed to reinforce the dental resin composites. Among lots of inorganic fillers, glass is the most usually used inorganic fibers during the reinforcement of dental composites. The following factors may interpret the reason why glass is most usually used. Firstly, the most essential demands of dental restoration composites could be satisfied by addition of appropriate amounts of glass. Secondly, glass possesses an index of refraction very close to that of the dental resins, equipping the resultant composites with semitransparent surfaces. Also, it is just for the reason of index of refraction mismatch with dental resins that crystalline SiO_2_ is unfitted to be reinforcing filler in dental composites. In general, when glass fillers in scale from dozens of nanometers to few microns are employed to reinforce the dental composites, the corresponding dental resin composites strengthen by micron-scaled glass fillers are unsuitable for broader uses under high stress circumstances, such as in case of replacement of cusps. Researchers have made a study about the enhancement of dental composites using certain glass fibers (with diameters of about ten microns) [24]; however, mainly because of poor interfacial adhesion, the mechanical properties of corresponding dental composites were indeed increased but finitely.

Both polymeric and inorganic nanofibers (with diameters of a few nanometers) can be fabricated feasibly and conveniently by means of established electrospinning technique, and we name these nanofibers “electrospun nanofibers” usually [25]. By way of electrospinning the amorphous silica and subsequently splitting at 800°C, glass nanofibers with diameters of 500 nm, homogeneous structure, and well-distributed morphology can be successfully obtained [26, 27]. It has demonstrated that when we substitute traditional dental glass filler with glass nanofibers partially, the strength and toughness of the corresponding resin composites will enhance greatly [28].

In terms of enhancement of dental composites, the nanoscaled glass fibers are believed to be so much better than conventional micron-scaled glass particles/fibers. It is the characteristics of electrospun nanofibers (such as petty diameter, huge surface area, tremendous aspect ratio, enormous strength, and modulus) that enable nanoscaled glass fibers to assume wanted structure, morphology, and performances. The following mechanism may explain the reason why small content of nanoscaled glass fibers can reinforce the dental composites drastically. When dental resin composites are under external pressure or three-body friction, microcracks are easily tended to be produced in the body of dental matrix. Although the cracks are formed, the gap between crack planes is not a vacuum and thin and long glass fibers exist in the middle section bearing load constantly, until glass fibers are broken completely. So, we can make an easy understanding that crack expansion is inhibited by the fibers, and meanwhile the matrix will become more powerful. In comparison with micron-scaled glass fibers, the nanoscaled glass fibers are thinner and also have substantial surface Si–OH groups which could interact with various silane coupling agents. Consequently, the interfacial adhesion force between the nanoscaled silanized glass fiber and the dental resin matrix will go more powerful.

In many of electrospun inorganic nanofibers, great attention has been given to glass nanofibers by researchers owing to well-understood pretreatment and extensive applications in various aspects [29]. Chen et al. [30] added electrospun glass nanofibers (with diameters of about 400 nm) into dental resin for strengthening and toughening. They concluded that electrospun glass nanofibers greatly excelled traditional glass fibers in both tension and impact tests as well as the enhancement in strength and toughness at content of 0.5% and 1%. In comparison with pure epoxy resin, the addition of electrospun glass nanofibers could raise the tensile strength, Young's modulus, and work of fracture by 12%, 33%, and 52%, respectively. Electrospun glass nanofibers with uniform morphology and irregular structure have been successfully fabricated by Chen et al., and even though glass nanofibers undergo violent ultrasonication, they can still maintain fiber morphology unchanged. Gao et al. [28] brought about the treated glass nanofibers into Bis-GMA/TEGDMA dental resin composites and subsequently confirmed the supposition whether the incorporation of glass nanofibers could cause tremendous enhancement on mechanical performances. Gao et al. demonstrated the small content substitutions of traditional dental filler with the treated nanoscaled glass nanofibers. The flexural strength and modulus improved obviously increased as much as 44% and 29%, respectively. In the end, Gao et al. hold the viewpoint that if only chemical constitution and treatment ways of nanofibers could be further optimized, the mechanical performances of dental resin composites would be increased to a higher level.

In addition, even if electrospun glass nanofibers suffer from powerful ultrasonic violence, the whole morphology of electrospun glass nanofibers could be well maintained indicating that the glass nanofibers possess both natural morphology and structure. So we have every reason to believe that the electrospun glass nanofibers can be the most promising candidate in the reinforcement of dental resin composites.

#### 2.1.2. Hydroxyapatite Nanofibers

Hydroxyapatite (HAP), tetracalcium phosphate, and dicalcium phosphate anhydrous are typical and biocompatible calcium phosphates fillers [31–36]. For releasing mineral into dental resin composites, calcium phosphates fillers above are generally added at some content. Also many researchers have shown that calcium phosphates are nontoxic [36]. Mechanical performances of dental resin composites could be reinforced significantly by means of introducing inorganic fibers with high toughness. Chen et al. divided the targeted dental resin composites into two groups, namely, Bis-GMA/TEGDMA dental resin composites containing silica filler at different loading content and without silica filler [37]. Moreover, the reinforcement of hydroxyapatite nanofibers with huge slenderness ratios on the targeted dental resins was seriously studied. By means of chemical approaches, hydroxyapatite nanofibers with slenderness ratios from 600 to 800 were smoothly fabricated. Owing to HAP nanofibers possessing sufficiently high slenderness ratios and relatively long and thin structure, a lot of bonding points could be created readily in the interface of nanofibers and other matrix. So in terms of mechanical performance, nanofibers are ideal reinforcing materials compared with other particles, since more stress transfer could be offered by interaction between nanofibers and matrix. Meanwhile, polymerization shrinkage could be cut down by the addition of HAP nanofibers. In brief, when the composites suffer from huge pressure, tremendous stress could be transferred from composites matrix to tough HAP nanofibers. Thus, substantial increase will be obtained in mechanical properties of dental composites finally. Chen et al. proved that biaxial flexural strength of corresponding dental resin composites could be improved drastically in the presence of hydroxyapatite nanofibers with different loading content. For instance, in terms of biaxial flexural strength, the dental resins without filling HAP nanofibers were at 100.3 MPa, and the dental resins with 10 wt% HAP nanofibers loading were at around 122.3 MPa. Obviously, biaxial flexural strength of filled dental resins raised by 22.2%. They drew a conclusion that when the loading content reached a certain point, mechanical performances of corresponding dental composites did not continue to increase any more with the growth of loading content, what is worse, some performances of corresponding dental composites would decrease greatly, for example, when the dental resins were loaded with HAP nanofibers at 40 wt%, the biaxial flexural strength will decrease to around 41.7 MPa. Hence, when such a situation above happens, we believe it has reached the percolation threshold of hydroxyapatite nanofibers. Finally, Chen et al. demonstrated that the biaxial flexural strength will be enhanced obviously at the condition of uniform dispersion of hydroxyapatite nanofibers, and the loading content is generally not too high. The improvement of dental resins composites will decline seriously, while loading content of hydroxyapatite nanofibers outnumbers percolation threshold. Since excessively high loading content of nanofibers prone to make reinforcing nanofibers into drawbacks and this will damage dental composites' mechanical performances greatly.

#### 2.1.3. Fibrillar Silicate

Lots of single crystals in level of nanometers make up fibrillar silicate. palygorskite, whose reserve is the largest in China and America, is the most rich fibrillar silicate minerals in all types of fibrillar silicate minerals in the natural world. Silicate single crystals (about 100–3000 nm in length and 10–25 nm in diameter) are the most basic components of fibrillar silicate and micron-scaled particles can be formed by stacking mentioned single crystals [38, 39]. Highly aligned structure and extraordinary mechanical performances are the greatest characteristics of single crystals. In terms of absolute strength, the single crystals in scale of nanometers (more than 50 GPa) are over ten times as high as that of most of microscaled fibers [38]. Although montmorillonite and fibrillar silicate are both composed of nanoscale units, the largest distinction between them is the different degrees of difficulty to totally segregate into nanoscaled silicate layers and to evenly spread in dental resin composites. A larger room among stacked single crystals in fibrillar silicate than that of montmorillonite may account for the reason why there is a difference between them. Just for the reason above, the interaction of silicate layers in montmorillonite is more powerful than that of silicate layers in fibrillar silicate. Consequently, just because of this microstructure of fibrillar silicate, we could easily split fibrillar silicate into nanoscale single crystals just by way of dissolution in polar solvent and physical stirring, and this easy separation contributes to distribute uniformly in the dental resins, thus enhancing the dental composites [39]. Besides, extremely rich Si–OH groups, which could have a good interaction with diversified silane coupling agents, exist on the surface of nanoscaled single crystals. Precisely because of existence of these Si–OH groups, the interaction between silanized single crystal exfoliated by fibrillar silicate and dental composites could be tremendously powerful. The silanization of nanoscaled single crystals could contribute to distribute uniformly in the dental matrix and make an increase on the mechanical performance accordingly. Specifically, when dental composites are under tremendous pressure suddenly, they tend to create cracks in the body of composites; meanwhile, fibrillar silicate single crystals could continue to function across the cracks and restrain the expansion of cracks. So the inhibition of crack-extending and increased interaction between single crystals and matrix result in the reinforcement on the final mechanical property of the resin. Moreover, the enhancement of Bis-GMA/TEGDMA dental resins with and without traditional filler was studied by Tian et al. [40]. Three dispersal approaches were employed to scatter nanoscaled single crystals into dental matrices evenly. Results showed that when the resin was loaded with 1 wt% nanoscaled fibrillar silicate, the modulus of elasticity, bending strength, and work of fracture increased by 16.7%, 40%, and 78.4%, respectively. Tian et al. drew a conclusion that the mechanical performances of dental resins/composites were improved tremendously when small content of nanoscaled single crystals was introduced into Bis-GMA/TEGDMA dental resins/composites. Also, the mechanical performances would not be enhanced and even would be damaged severely when greater content of nanoscaled single crystals was introduced. However, it is very difficult to distribute nanoscaled fibrillar silicate into dental composites evenly. In a word, an enhanced effect or weakened effect almost completely depends on the dispersion of nanofibrillar silicate into dental composites.

### 2.2. Reinforcement of Polymeric Nanofibers

#### 2.2.1. Electrospun Nylon 6 Nanofibers

The most commonly used method to prepare kinds of ultrafine nanofibers is electrospinning because of its convenience and simplicity [41–44]. The basic principle of electrospinning can be described in the following way: the polymer solution drop was charged in the electric field, and electrical force drives the spray of polymer solution drop via a syringe needle.

In the course of preparing nylon 6 nanofibers by electrospinning [45], accelerated drawing in an irregular way enables polymer solution drop to stretch astonishingly in a very short time (about elongation of 100,000 times in less than 1/10 s). It is because of such substantial draw that conformations of polymer chains and microstructure of polymer are altered. Hence, the strength of the polymer nanofibers prepared by electrospinning will be very powerful. At the same time, the interfacial linking force between nylon 6 nanofibers and dental matrix will be very solid for the reason of high specific surface area of nanofibers. As everyone knows, the intensity of interfacial linking force between nanofillers and matrix has a decisive influence on the strength of overall dental composites. Therefore, we believe that the nylon 6 (diameters in the range from 100 to 600 nm) nanofibers could enhance the dental resin composites. The enhancement of nylon 6 nanofibers on Bis-GMA/TEGDMA dental composites was investigated by Fong [46]. They dissolved the nylon 6 nanofibers into hexafluoroisopropanol solution and obtained nanofibers by electrospinning. The tiny size of nanofibers possesses high specific surface area which may increase the interfacial adhesion between nylon 6 nanofibers and the resin matrix and lead to the improvement of performances of dental resins to a higher level. Fong also proved that the microcracks will be prevented efficiently in the presence of nanofibers. They found lots of fracture lines which were initiated in the course of fracture process on the break surface. These phenomena show that when nanofibers are arrayed along the fracture direction, it will consume huge amounts of energy in the course of complete tear. In other words, when high pressure force was imposed on the dental composites, owing to powerful forces between nylon 6 nanofibers and dental matrix, it will be extremely hard to form microcracks firstly, even if microcracks appeared, nylon 6 nanofibers will prevent the expansion of cracks. What is more, lots of energy will be consumed in the course of being torn for nanofibers, thus leading to the substantial improvement of resin composites' FS and work of fracture. However, they found no existence of residual resin in the terminal of extracted nanofibers by SEM. Little existence of residual resin demonstrates interfacial linking force between the BIS-GMA/TEGDMA dental resin and nylon 6 nanofibers that remain to be enhanced for boosting dental restorative composite. Tian et al. [47] considered that addition of fibrillar silicate (with well distribution and high arrangement) into nylon 6 nanofibers could enhance the whole mechanical performances of composite nanofibers, and the impregnation of the composite nanofibers could reinforce the dental composite efficiently. Tian et al. demonstrated that the mechanical performances of dental resin composites will be raised tremendously via small content of addition of composites nanofibers. Nevertheless, wanted reinforced effect does not appear in the case of larger content of addition of composites nanofibers. They considered that existence of flaws inside the matrix, finite mechanical performances of composite nanofibers, and low strength of interfacial linking in the interface of nanofibers and the dental matrix were main reasons. What is more, when dental resin composites are under high loading environment, microcracks will be created in the body of composites, and the nanofibers exist in the middle of cracks planes sustaining the heavy burden owing to the continuation of electrospun nanofibers. Consequently, continuous nanofibers play a critical role in inhibition of crack growth. For the purpose of acquiring ideal enhanced effects, electrospun nanofibers with high alignment rather than random distribution are needed. In addition, Dodiuk-Kenig et al. [48] studied the enhancement of electrospun nanofibers on the dental composites with the modification of hyperbranched polymer. The appearance of new functionalized nanofillers opens a door to the formation of the latest composites and the substantial increase in the mechanical performances of dental composites. Dodiuk-Kenig et al. discussed the reinforcement of nylon 6 nanofibers with different contents and different surface treatment on dental resin composites, and they considered that the reinforcement lies in the close linking between resin matrix nanofibers. Similarly, bad performances connect with poor interfacial adhesion. And it can be completely proved by the enhancement of treated nanofibers and untreated nanofibers.

#### 2.2.2. PAN-PMMA Nanofibers

**Figure 2:**
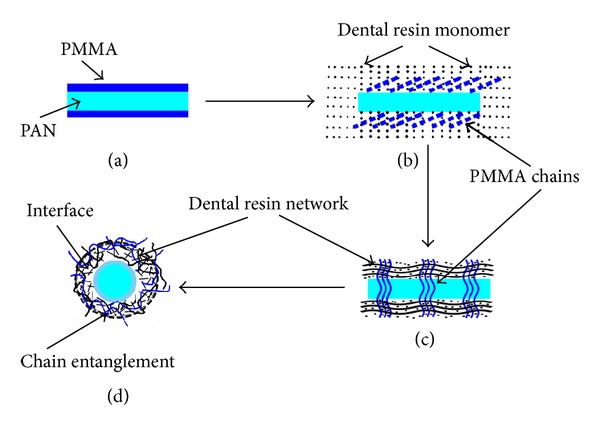
Diagram of formation of composites' partial interpenetrating network. (a) Nanofibers with core-shell structure. (b) Nanofibers surrounded by resin matrix. (c) Formation of partial interpenetrating network. (d) Formation of tanglement between PMMA and dental resin in the interface.

Variety of nanofibers can be fabricated successfully by means of electrospinning technique. Generally speaking, nozzle is connected with positive electrode, and the collector is usually linked with cathode. The polyacrylonitrile/poly (methyl methacrylate) (PAN/PMMA) membrane was smoothly prepared by electrospinning by Lin et al. [49]. By way of the characterization of transmission electron microscope, they confirm the PAN-PMMA nanofibers (with an outer diameter about 290 nm) and the result of an energy dispersive spectroscopy is in accord with above claim. The formation of partial interpenetrating network in the interface of nanofibers/matrix produces drastically powerful interfacial linking force which functions mainly in the enhancement of composites (Figure 2).

Some shortcomings still exist in nonwoven nanofibers (diameters in the range of 500–800 nm) as reinforcing filler in spite of a few achieved accomplishments. Report said that irregular alignment of nanofibers leads to a decreased crystallinity and thus influencing the intensity of nanofibers membranes seriously [50]. At the same time, irregular alignment of nanofibers caused by their excessive pore and bad orientation will cause the enhancement effect to fade [51].

Lately, for the sake of raising the strength of electrospun nanofibers, much attention has been focused on the poststretching handling of nanofibers (diameters in the range of 300–500 nm) [52, 53]. It is considered that the extent of crystallization and regular alignment directly determines the properties of nanofibers itself. Also, that extent could be raised by the poststretching handling. For satisfying the higher requirements in terms of mechanical performances, the PAN/PMAA nanofibers with special structure and corresponding dental resin composites were investigated by Sun et al. [54]. A cylinder rod with high-speed rotation was employed to collect the electrospun nanofibers, thus acquiring nanofibers membranes arranged in parallel. After that, poststretching handling is a great choice to raise the extent of crystallization and regular alignment and then increase the strength of nanofibers accordingly. Sun et al. showed that the tensile performances and the extent of parallel alignment in nanofibers membranes were enhanced obviously via poststretching handling. The mechanical performance of Bis-GMA/TEGDMA dental composites was boosted indeed with the impregnation of PAN-PMMA nanofibers. For instance, with the increase of nanofibers content within certain limits, the mechanical performance of dental resin composites, such as flexural modulus and strength, maintains growing. Compared with untreated nanofibers, the reinforcement of poststretching nanofibers on dental composites is much better, especially in the terms of flexural performances. Moreover, better interfacial linking force exists in the dental resin enhanced by poststretching nanofibers via examining break surface using scanning electron microscopy.

So, we can completely consider that mechanical performance (especially tensile properties) of electrospun nanofibers membranes can be enhanced clearly by the process of poststretching treatment. Furthermore, compared with normal electrospun PAN/PMMA nanofibers, dental composites strengthened by poststretching nanofibers possess better mechanical performance (such as flexural properties and fracture energy). Ultimately, Sun et al. believed that formation of partial interpenetrating network in the interface and enhanced strength of poststretching nanofibers are the biggest reason why the mechanical performances (especially tensile properties) of Bis-GMA dental composites can be intensified by poststretching PAN/PMMA nanofibers with special structure.

#### 2.2.3. Other Nanofibers

By means of electrospinning which is a convenient, simple, and practical technique, we can prepare various nanofibers in the scale from some micrometers to nanometers (generally less than 500 nm). But, owing to relatively low mechanical properties of natural nanofibers, they are usually not used as fillers to reinforce dental composites [55–60]. Through the development of recent decades, electrospinning technique has acquired substantial progress, and the corresponding nanofibers have been applied to lots of fields. Besides nylon 6 nanofibers and polyacrylonitrile/poly methyl methacrylate nanofibers, electrospun polyvinyl alcohol (PV–OH) nanofibers could also boost the mechanical performances of dental composites. Dodiuk-Kenig et al. [48] investigated the enhancement of PV–OH nanofibers (with a diameter of 250 nm and aspect ratio of about 1000) on the dental formulations modified by other special polymers. They regarded PV–OH nanofibers as an enhancement factor into the composites, and added the enhancement factor with and without surface treatment into the composites with a very small content. In Dodiuk-Kenig's paper, they character the corresponding dental composites enhanced by PV–OH nanofibers via several methods, and these characterizations (especially SEM images of composites section) facilitate our understanding on the mechanism of reinforcement of PV-OH nanofibers on dental composites. In a word, they considered the reinforcement consisted in the tight linking between resin matrix and nanofibers. Similarly, bad performances connect with poor interfacial adhesion, and it can be completely proved by the enhancement effect of treated nanofibers and untreated nanofibers. They concluded that addition of 0.05 wt% PV–OH nanofibers with a diameter of 250 nm into composites could increase the compressive strength by 30% and decrease the shrinkage by 50%. While little enhancement can be obtained by nanofibers with another diameter, what is worse, the addition of nanofibers with another diameter even brought about a loss of performance.

As we know, polymer nanofibers take on lots of advantages, such as wide range of scale, distinctive structure, super-high specific aspect ratio, and outstanding mechanical performances. Selection of diverse polymer species, mix of different polymers, various treatment ways of nanofibers, and other factors could have a substantial influence on the structure, composition, and performance of electrospun nanofibers. By electrospinning technique, we enable sorts of nanofillers (such as kinds of inorganic nanoparticles) to impregnate the body of composites uniformly. For example, we could put multiwalled carbon nanotubes (MWCNT) to dissolve in the particular polymer solutions, and then we could obtain continuous nanofibers containing carbon nanotubes which can be used as nanofiller by electrospinning technique. Chronakis [61] successfully fabricated MWCNT-PAN nanofibers using electrospinning. Nanofibers with special properties also could be achieved via all kinds of advanced preparation technology [62, 63]. Additionally, natural polymer is also a good candidate reinforcing filler; for example, some researchers made a deep study about reinforcement of collagen-based scaffold chitin nanofibers for bones [64].

The simplicity of modification on both surface and internal of polymer nanofibers is another reason why we focus on the polymer nanofibers [65]. Various functionalizations of polymer nanofibers after the synthesis are easy to realize. Moreover, for preparing nanofibers with special structures and morphology, it is very likely to regulate and control the structures of nanofibers as we designed [66]. Consequently, polymer nanofibers are considered to be ideal candidate nanofillers in reinforcing the mechanical performances of dental composites.

### 2.3. Ceramic Nanofibers

Despite dental resin composites are broadly applied to restore tooth. The service life of composites is far shorter than that of amalgams because of inner cracks and fracture [67]. In order to raise the mechanic performances and decrease inner cracks and fracture, many kinds of special fibers have been employed to increase overall strength of composites. However, most of these materials are unsuitable because of behavior, stabilization, and aesthetic requirements. It is reported that composites boosted by glass nanofibers assume weaker mechanical performances after absorbing water [68]. Ceramic materials possess many advantages, such as outstanding physical performance, stabilization in high temperature, and excellent compatibility. By means of fusing, we acquire ceramic fillers in the form of single crystal whiskers, and the existence form of whiskers favors the better silanization of themselves. In general, ceramic fillers possess huge aspect ratio (with a diameter and length of approximately 0.5 mm and 5 mm, resp.) [69, 70]. Moreover, ceramic nanofillers are in the form of single crystal whiskers which take on a tremendous tensile strength at about 50 GPa compared with glass fibers at about 3 GPa [71]. The small size and relatively excellent structure of ceramic whiskers could provide a sensational distribution in the dental matrix, which facilitates the increase of interbonding between ceramic whiskers and matrix. Just because of various advantages of single crystal whiskers above, when dental composites suffer from enormous pressure, ceramic whiskers have a great potential to inhibit the emergence of microcracks and prevent its enlargement. That is to say, huge external pressure will be largely resisted by ceramic whiskers, which contributes to greatly increase the mechanical properties of the resin composites. Also, it was said that the strength and toughness of thermal-curing composites doubled with suitable addition of tremendously strong Si_3_N_4_ and SiN ceramic whiskers [72–74]. Since there exists mismatch between whiskers' refractive and resins' refractive, it will restrict composites' application severely. Some ceramic nanofibers, such as zirconia-yttria-silica, zirconia-yttria, and zirconia-silicia, can be successfully obtained by Xu et al. by electrospinning way and following sintering [75]. Particularly, due to slippery surfaces and special configuration of zirconia-silicia and zirconia-yttria-silica nanofibers (with diameters from 100–300 nm), it is very possible to apply them in the enhancements of dental composites. The enhanced effect of zirconia-silicia and zirconia-yttria-silica on dental composites was studied by Guo et al. [76]. The reason why they choose zirconia-silicia or zirconia-yttria-silica as addition to enhance composites is because of their super-high strength, excellent stabilization, biocompatibility, and low light scattering. Heat curing and light curing are applied in the course of fabricating the control composites and experimental composites. Finally, they drew a conclusion that overall properties could be increased obviously (such as flexural strength and modulus and fracture energy) with impregnation of zirconia-silicia and zirconia-yttria-silica nanofibers at content of 2.5% and 5.0%, respectively. If they continued to raise the amount of zirconia-silicia nanofibers up to 7.5%, reversely, main performances decreased clearly. All dental composites enhanced by zirconia-silicia possess obviously higher fracture toughness than the control. In order to put the strength of dental composites to a completely new level with increasing nanofibers, we need new-fashioned and effective nanofibers. Consequently, other ceramic nanofibers and smaller content of zirconia/silica are being studied. Of course, we should try to work out innovative ways to make the mechanic performances of the composite stronger and their service life longer, and this is the long-term target that we have been pursuing.

## 3. Reinforcement of Nanotubes

### 3.1. Inorganic Nanotubes

#### 3.1.1. Halloysite Nanotubes

In terms of strength and service life, dental amalgams are far better than that of composites enhanced by majority of inorganic fillers. The applications of dental composites strengthened by much of inorganic fillers are restricted seriously just because of reason above [77–79]. Currently, the dental composites on the market can only meet essential demand of filling tooth but fail to be employed in high pressure-assuming positions. Moreover, in contrast to the life of dental amalgams, the service life of dental composites is one-third of that of dental amalgams. The mechanical intensification of dental composites could be boosted drastically by means of addition of fibers/whiskers with micrometer scale [80, 81]. For instance, Xu et al. [82] thought that the mechanic strength and toughness of dental composites doubled when certain content of tremendously strong ceramic fibers/whiskers was added into composites. Moreover, the ability to polish and handle was improved partially [83–85].

Being different from fibers in scale of microns, nanoscaled crystals make up hallosite [84]. Halloysite is a very commonly used natural mineral as a result of halloysites' wide source, simple purification steps, and high cost performance [85]. The length of halloysite nanotubes (with diameters of dozens of nanometers) are generally in the scope from 200 nm to 1 *μ*m and Young's modulus of halloysite nanotubes is in the scope from 230 to 340 GPa. Moreover, safety, biocompatibility, and easy handling are also the characteristics of halloysites. Compared with montmorillonite, halloysite is more easy to exfoliate into single halloysite nanotubes (HNTs) and to scatter in the dental composites evenly.

The room among stacked layers may account for the reason. The room among silicate layers in montmorillonite is smaller than that of hallosite; hence, the interaction of montmorillonite is more powerful than that of hallosite. Hence, as to hallosite, it is not necessary to replace metal ions with special reagent at all. The halloysite nanotube in halloysite can be split fully in the presence of polar solvents and mechanical agitation. Moreover, as a result of existence of rich Si–OH group on the external of halloysite nanotubes (HNTs), the silanized halloysite nanotube could be closely connected with dental resin matrix via tremendously powerful interfacial linking force. The enhancement of Bis-GMA/TEGDMA dental resins or composites boosted by various contents of HNTs was studied by Chen et al. [86]. For splitting the halloysite into halloysite nanotubes and scattering evenly in dental composites, three approaches were employed. They came to a conclusion that introduction of low content of the silanized HNTs into dental resins/composites brought about a clear enhancement. But, high content of addition did not cause improvement on the mechanic performances. Briefly, the reinforcement of halloysite nanotubes almost entirely depends on the dispersion effect in the matrix. Excellence dispersion facilitates reinforcing the dental resins/composites; halloysite nanotubes are strengthening phase. On the contrary, halloysite nanotubes will become harming phase, thus damaging the overall performance of dental resins/composites. For the mechanical performances of dental resins/composites could increase tremendously by the addition of well-distributed halloysite nanotubes at appropriate content. Halloysite nanotubes have huge potential to be effective strengthening filler in enhancement for dental composites.

#### 3.1.2. Carbon Nanotubes and Boron Nitride Nanotubes

As everyone knows, carbon nanotubes possess astonishing mechanical performances and excellent biocompatibility [86–88]. For instance, the modulus of carbon nanotubes is ten times stronger than that of steel; exactly, single-walled carbon nanotubes (SWCNTs, with a high aspect ratio at around 500–1000) possess outstanding mechanical performance. For instance, it takes on a tensile strength at 50–100 GPa which is five times higher than that of steel, but the weight of the steel is six times than that of carbon nanotubes [89–93]. Given the remarkable physical performances and ultrahigh specific surface area, single-walled carbon nanotubes are really very promising in the reinforcement of dental composites and very likely to replace glass fiber fillers. Single-walled carbon nanotubes have been used for hard-tissue application, such as the regeneration and repair of bones and teeth, and have a good biocompatibility in general case [94, 95]. The improved effect of dental composites is restricted for the reason of carbon nanotubes' gathering and weak interfacial linking with composites. Nanotubes prefer gathering together and the condition of nanotubes' distribution in polymer solution becomes extremely important for enhancement [96]. Generally, nanotubes are prone to agglomeration, so it is very difficult to disperse evenly. Moreover, the surface of nanotubes is so smooth that it will affect the interfacial adhesion force between the nanotubes and composites. If agglomeration happens, the nanofillers will be drawn out from the dental composites instead of breaking and nanotubes' function is restricted to large extent. Zhang et al. [95] gained a well-distributed effect in the dental composites by way of coating single-walled carbon nanotubes with modified nano-SiO_2_. Eventually, what we hope is excellent scattering and substantial bonding force of single-walled carbon nanotubes after addition into the dental composites, and we also expect that the performances could be enhanced tremendously. The impregnation of modified single-walled carbon nanotubes indeed brings about enhancement of dental resin composites obviously by the statistical analysis. Moreover, other researchers indeed demonstrated that the carbon nanotubes could be a very promising scaffold material for dental materials [97].

Boron nitride nanotubes, which possess tremendous tensile strength, have a similar structure to carbon nanotubes [98]. Exactly, they exhibit extraordinary tensile strength at about 30 GPa and Young's modulus at about 900 GPa; moreover, the oxidizing temperature may reach as high as 800°C. Boron nitride nanotubes gained so much attention because of its ultrahigh performances in strengthening effect. By the means of hot pressing, BNNTs/alumina composites were prepared by Wang et al. [99] and the reinforced effect of Boron nitride nanotubes on the mechanic performances of alumina matrix was studied. They concluded that the flexure strength would be raised with the introduction of Boron nitride nanotubes at higher temperature.

### 3.2. Metallic Nanotubes

*Titania Nanotubes.* The appearance of nanomaterials provides an opportunity to advance the mechanical performances of dental composites and bone cements to a new level [100, 101]. Khaled et al. gained a very good enhancement effect of poly methyl methacrylate by way of addition of titania nanofibers [102]. Nanotubes (average diameter is 200 nm) have a high ratio of length to diameter and super-high specific area as good as nanofibers. Precisely because of above characteristics, it is very possible to heighten the physical performances obviously. Furthermore, the effect of extra intertwining (both internal and external interlinking of nanotubes) can be obtained entirely due to tubular structure of titania nanotubes. Khaled et al. [103] carried out a research for the purpose of boosting the physical performances of common acrylic cement via addition of late-model titania nanotube which acts as a strengthening phase. The main purpose is to procure the enhancement of dental cements via the addition of strengthening phase and compare with enhanced effect of current fillers. And they concluded that pressure could be transformed easily and efficiently via extremely powerful interface, because there exists powerful interfacial adhesion between treated TiO_2_ nanotubes and dental matrix. Certainly, this late-fashioned nanofiller coupled with the current fillers could be employed together to strengthen flowable and usual dental composites with no damage in biocompatibility.

Generally speaking, the nanofibers take on the shape long and thin and possess diameters at hundreds of nanometers. Nanotubes have the tubular structure and slightly bigger diameters. As with surface properties, lots of functional groups exist on the surface of nanofibers, such as –OH group, but these groups do not exist on the nanotubes commonly, so we should conduct a treatment on the nanotubes before their usage. Owing to distinctive structure of nanotubes, they usually have relatively high strength than nanofibers, but it does not mean that nanotubes are better fillers than nanofibers. For reinforcement of dental composites, it mostly depends on the condition of nanofillers themselves and their status in the matrix, such as dispersion, interbonding, and other influence factors. As long as we take full and reasonable advantage of these nanofillers, the substantial increase on mechanical performance of dental composites will be acquired definitely [104].

## 4. The Potential Reinforcement Mechanism

Through the study of so many cases of enhancement of nanotubes/nanofibers, a reinforcing mechanism could be seen clearly. Namely, under high external pressure, microcracks tend to be created in the body of dental composites. However, the room of cracks is not empty, nanofibers or nanotubes still exist in the middle of cracks plane and function across planes. Thereby, cracks are prevented via the remaining fillers. Hence, much of the stress is transmitted through the fillers into the resins, and that is the reason why the nanofillers must be largely tougher than the resin matrices. Of course, the Van der Waals' force via physical entanglement and chemical bonding between nanofillers and matrix lays the theoretical foundation for the improvement of dental composites' performance. The enhanced performances of composites boosted by poststretching PAN/PMMA nanofibers are the best example of previous viewpoint. So, powerful mechanical performances and ultrasmall scale are the necessary condition for valid bridging fillers.

Another strengthening mechanism is that uniform dispersion of fillers throughout the matrices is needed. Supposing that the immersed parts of fillers are irregular in appearance, it will tend to form the stress concentration easily. Also, stress concentration is the main reason for creating small cracks in the matrix, thus damaging the properties of composites. Therefore, to produce the biggest effect of chemical bond, nanofillers should be dispersed as uniform as possible. The nanofillers which are highly aligned along some orientation (such as across the cracks) could impede the growth of crack, thus raising the mechanical behavior of dental resin and reinforcing the dental composites.

Last strengthening mechanism but most importantly is the interaction between nanofillers and composites. In the course of preparing composite materials, what we should consider most is the interactions between fillers and resin matrix, namely, interfacial adhesion. In the condition of filler-strengthened dental composites, interfacial properties have a crucial impact on the performances of resulting dental composites. High fillers surface area could raise the extent of entanglement between various fillers and surrounding dental resin and the best case is the formation of the mutual permeating structure in the middle of fillers and the dental matrix, which bring about a specific interface in the nanometer scale. At the same time, powerful interfacial binding force between different phases aimed to hinder the movement of polymer chains is prospected to be produced, and then corresponding mechanical performances of resultant dental composites will be reinforced. For the purpose of enhancing the interfacial chemical binding, various measurements will be taken, such as functioning of nanofillers. As to this strengthening mechanism, extremely strong chemical bond between nanofillers and matrix is the most vital factor for reinforcing.

**Figure 3:**
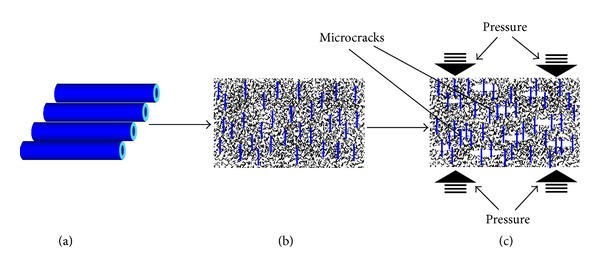
Diagram of reinforcement mechanism of dental composites. (a) Extremely hard nanofibers or tubes, (b) extremely hard nanofibers/tubes highly aligned along some orientation uniformly in the dental composites, and (c) when dental composite subjected to powerful pressure, nanofibers, or tubes acts as a bridge across the microcrack which impedes the growth of crack, and then dental composites will be reinforced.

So, in my opinion, the potential reinforcement mechanism is that extreme toughness nanofibers and nanotubes are fabricated. Provided that immersed parts of fillers are irregular in appearance, it will tend to form the stress concentration easily. Also, stress concentration is the main reason for generating small cracks in the matrix, thus damaging the properties of composites. Then we disperse them uniformly in the dental matrix in some direction and when the microcrack emerges. Powerful bonding between nanofibers and nanotubes will impede the growth of cracks, which will greatly facilitate the reinforcement of dental composites (Figure 3). In brief, no matter what treatment you conduct on the surface of nanofillers, chemical bond between nanofillers and matrix is always to be considered firstly. Only by means of the formation of strong chemical bonding in the composites can you could obtain reinforced dental composites. Finally, on the basis of powerful chemical bonding, we could increase Van der Waals' force electric attraction as far as possible.

## 5. Conclusions and Perspectives

In this review, the efforts using nanofibers and nanotubes to reinforce mechanical performances of dental composites, including polymeric nanofibers/nanotubes, metallic nanofibers, and inorganic nanofibers/nanotubes, as well as their researches related, are demonstrated in sequence. We demonstrated the use of kinds of nanofibers or nanotubes to reinforce dental composites. Addition of nanofibers or nanotubes could acquire composites materials with distinct performances in lots of areas. Nanofillers are very different from traditional fillers largely because of their scale in nanometers and special microstructure, and we can make a substantial increase on the mechanic properties of resin composites by means of nanotechnology. On the way to conquer current flaws of traditional dental composites, fillers in forms of nanofibers or nanotubes are the most promising fillers to raise the mechanical performances of dental composites tremendously at a low content. We also make a general understating of the potential reinforce cement mechanism, the reinforcement effect which greatly depends on nanofillers' toughness, condition of nanofillers' dispersion in the body, and powerful bonding between nanofillers (in forms of nanofibers or nanotubes) and matrix. It is because we already have a broad understanding of the influence factor of reinforcement effect that we have a clear direction for the future development.

In the future, a further study on the mechanism of reinforcing the dental composites should be carried on, as well as the influence factor of reinforcement effect. Only when we master the mechanism of reinforcing the dental composites well and the influence factor of reinforcement effect can we make a further increase reinforcement of the dental composites. Additionally, more novel methods of preparing nanofibers or nanotubes will be introduced. In order to put the mechanical performances of dental resin composites to a completely new level, more tough nanofillers should be fabricated, the method of dispersing nanofillers more uniformly should be found, and interfacial adhesion between fillers and the body should be more and more powerful.
